# A Case of Acute Necrotizing Encephalopathy With Multiple Organ Failure Following COVID-19

**DOI:** 10.7759/cureus.51665

**Published:** 2024-01-04

**Authors:** Naohiko Maejima, Shotaro Matsumoto, Itaru Hayakawa, Kentaro Koike, Yuichi Abe

**Affiliations:** 1 Division of Critical Care Medicine, National Center for Child Health and Development, Tokyo, JPN; 2 Division of Neurology, National Center for Child Health and Development, Tokyo, JPN

**Keywords:** cytokine, therapeutic plasma exchange, intravenous methylprednisolone, covid-19, multiple organ failure, acute necrotizing encephalopathy

## Abstract

Neurological complications are frequent non-respiratory complications associated with coronavirus disease 2019 (COVID-19), and acute encephalopathy (AE) has been reported to occur in 2.2% of patients. Among many phenotypes of AEs, acute necrotizing encephalopathy (ANE) is associated with multiple organ failure (MOF), leading to severe neurological morbidity and mortality. A previously healthy seven-year-old girl presented with a one-day history of fever followed by 12 hours of vomiting and altered consciousness. On arrival, the patient was in shock. Blood tests revealed severe acute liver failure and kidney injury, accompanied by coagulopathy. The serum interleukin-6 levels were also elevated. PCR testing for severe acute respiratory syndrome coronavirus 2 (SARS-CoV-2) was positive. A head CT scan showed heterogeneous low-density areas in the bilateral thalamus, without brainstem involvement. She was diagnosed as ANE complicated with MOF (ANE severity score = 6). Intravenous methylprednisolone and therapeutic plasma exchange (TPE) were initiated with neurocritical care. After the introduction of TPE, hemodynamics improved rapidly, followed by gradual improvement in neurological manifestations. Upon follow-up after two months, no neurological or systemic sequelae were noted. Although further studies are needed, our case suggests that early immunomodulatory therapy and TPE may have contributed to the improvement in ANE and MOF associated with COVID-19.

## Introduction

Coronavirus disease 2019 (COVID-19) has spread rapidly worldwide since it was first reported in 2019. Neurological complications are frequent non-respiratory complications associated with COVID-19, and acute encephalopathy (AE) has been reported to occur in 2.2% of patients [1]. Acute necrotizing encephalopathy (ANE) is one of the most severe forms of acute encephalopathy, accounting for approximately 4% of all AE cases. To date, only a few cases of pediatric ANE with COVID-19 have been reported; however, the majority of cases have resulted in devastating neurological sequelae [2-4]. Here, we report a case of severe ANE associated with COVID-19 with favorable outcomes after early immunomodulatory therapy and therapeutic plasma exchange (TPE).

## Case presentation

**Figure 1:**
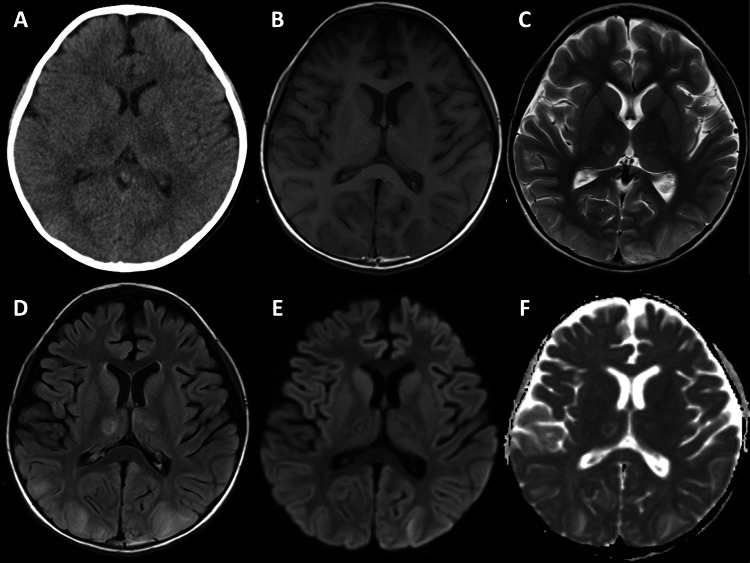
Imaging studies (A) day 2 CT scan, (B) day 16 T1-weighted image, (C) day 16 T2-weighted image, (D) day 16 fluid-attenuated inversion recovery, (E) day 16 diffusion-weighted image, (F) day 16 apparent diffusion coefficient map

A previously healthy seven-year-old girl presented with altered consciousness and frequent episodes of vomiting over 12 hours following fever (maximum 41.5 degrees Celsius for 40 hours). The patient was not vaccinated for severe acute respiratory syndrome coronavirus 2 (SARS-CoV-2). The patient was diagnosed with COVID-19 based on the antigen test. At the time of the visit, the patient was comatose with a Glasgow Coma Scale (GCS) score of E3V2M4. Blood tests revealed hepatic and renal dysfunction and mild coagulopathy (aspartate aminotransferase (AST) 92 U/L, alanine transaminase (ALT) 66 U/L, lactate dehydrogenase (LDH) 456 U/L, blood urea nitrogen (BUN) 20.5 mg/dL, creatinine (Cre) 0.69 mg/dL, platelet 214,000/µL, prothrombin time-international normalized ratio (PT-INR) 1.63, and activated partial thromboplastin time (APTT) 38.4 sec). Blood pressure gradually decreased to 66/38 mmHg, with impaired peripheral perfusion. The patient did not respond to 20 mL/kg crystalloid infusion; therefore, she was transferred to a tertiary hospital for advanced care. The patient’s consciousness level was E3V5M6 on arrival. The PCR test results for SARS-CoV-2 were positive. A CT scan showed a heterogeneous low-density area in the bilateral thalamus while the corticomedullary boundary was well preserved. No brainstem lesion was present (Figure 1).

**Figure 2:**
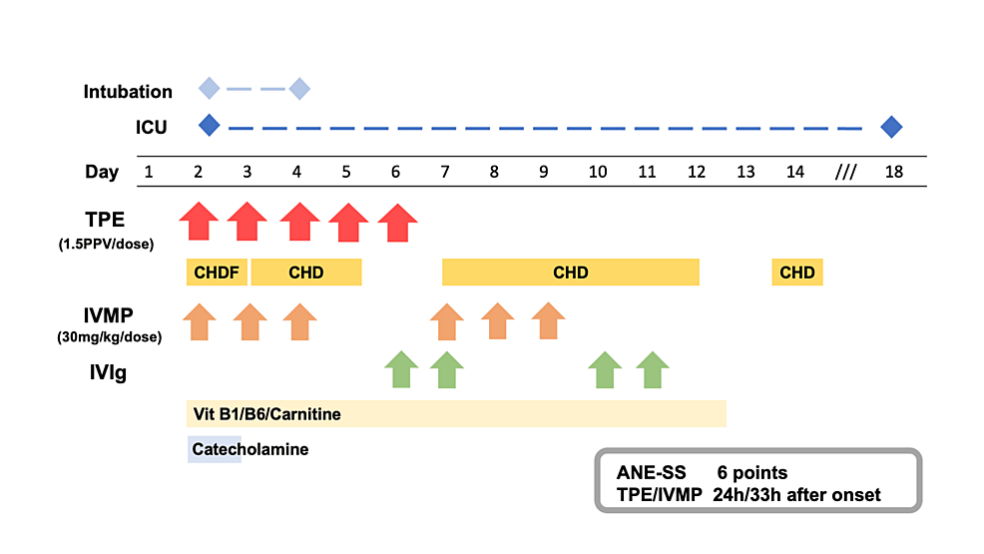
The clinical course of the patient The days of illness were defined by the day when altered consciousness was observed (onset of encephalopathy. TPE, IVMP, and IVIg are indicated by red, orange, and green arrows, respectively. Abbreviations: ANE-SS, acute necrotizing encephalopathy severity score; CHDF, continuous hemodiafiltration; CHD, continuous hemodialysis; IVMP, intravenous methylprednisolone; IVIg, intravenous Immunoglobulin; PPV, predicted plasma volume; TPE, therapeutic plasma exchange

The patient was transferred to our pediatric ICU due to shock, deteriorating hepatic and renal function, and progressive coagulopathy (AST > detection limit, ALT 10,800 U/L, LDH 10,577 U/L, BUN 48.7 mg/dL, Cre 1.26 mg/dL, platelet 83,000/µL, PT-INR 2.59, APTT 38.4 sec). On arrival at our hospital, vital signs were body temperature 37.2 ℃, heart rate 118 beats/min, blood pressure 110/85 mmHg, respiratory rate 20/min, percutaneous oxygen saturation 97% (room air), and GCS E3V4M6. Blood tests showed severe liver and kidney injury and coagulopathy, and echocardiography revealed poor cardiac contraction. A cerebrospinal fluid (CSF) examination could not be performed because of shock and severe coagulopathy. She was diagnosed with ANE complicated by multiple organ failure (MOF) based on the clinical course, laboratory tests, and CT findings. The ANE severity score (ANE-SS) was 6 points. The patient was intubated, resuscitative fluid was administered, and administration of inotropes and vasopressors was initiated. In addition to neurocritical care, a course of three-day intravenous methylprednisolone (IVMP, 30 mg/kg/day) and a course of five-day TPE to remove proinflammatory cytokines were introduced 24 hours after onset (Figure 2). Simultaneous continuous veno-venous hemodiafiltration was initiated for the acute kidney injury. Due to the severe liver and kidney injury, antiviral drugs for COVID-19 were not administered.

Following the introduction of the first TPE (1.5 plasma volume replaced with fresh frozen plasma over 6 hours), the patient’s hemodynamics markedly stabilized. The inotropes and vasopressors were quickly reduced and stopped the following day. Although fluctuating, the motor component of GCS remained at M6. On the other hand, although continuous EEG initially showed alpha waves predominantly in the occipital region and no slow waves, it started to show monomorphic delta waves on day 3, followed by gradual improvement from day 6. No electroencephalographic seizures were noted. A CSF test performed on day 3 revealed a mildly elevated cell count (21/µL) and CSF protein level (59 mg/dL). The CSF was negative for SARS-CoV-2 by PCR and bacterial culture. The patient was extubated on day 3 of admission. Following the completion of the five-day TPE, intravenous immunoglobulin (IVIG, 2 g/kg over 24 hours) was administered, and the second course of three-day IVMP was started from day 7 because her level of consciousness was still periodically fluctuating and EEG showed intermittent high-voltage slow waves. Serum interleukin (IL)-6 before TPE was elevated to 59.6 pg/mL but had decreased to < 1.5 pg/mL by day 9. The CSF IL-6 level on day 3 was elevated at 37.2 pg/mL. Serum and CSF IL-6 were not measured on the same day. Head MRI on day 16 demonstrated symmetric high intensity in a T2-weighted image of the bilateral thalamus (mainly in the ventral posterior complex), compatible with ANE, as well as occipital and parietal cortical and subcortical regions (Figure 1). She required renal replacement therapy until day 14 and was transferred to the general ward on day 18. The patient was discharged from the hospital on day 38 of admission without any neurological sequelae. At a follow-up visit to the neurology outpatient department, two months later, the patient was attending school and was fully functional without any residual disability in the motor and intellectual aspects of daily living.

## Discussion

We have presented the detailed clinical course of severe COVID-19-associated ANE complicated by MOF. To the best of our knowledge, this is the first case of a patient with ANE plus MOF who was discharged with no neurological or systemic sequelae.

Multiple neurological manifestations of COVID-19 have been described, ranging from mild symptoms, such as headache and dizziness, to more severe phenotypes, including stroke and encephalopathy. A recent large multicenter cross-sectional study in the US (n=15,137) demonstrated that 7.0% of children hospitalized with COVID-19 had concurrent neurologic complications, among which 2.2% was encephalopathy [1]. A prospective cohort study in the UK (n=1,334) found that 3.8% of children hospitalized with COVID-19 had neurological manifestations, of which 2.0% had severe encephalopathy or encephalopathy with no specific mechanism identified [5]. A nationwide survey in Japan identified 31 patients with acute encephalopathy (ANE, n=2) [6]. In this study, 9 (29.0%) had severe sequelae or died, which was more frequent compared to those with other viruses in the previous report [7].

ANE is one of the most devastating types of infection-triggered encephalopathy syndromes, with poor prognosis, including death (28%) and severe neurological sequelae (56%) [8]. Influenza (41%) was the most common causative virus, followed by human herpesvirus 6 (20%). Shock, age >48 months, presence of brainstem lesions, low platelet count, and CSF protein level >60 mg/dL have been associated with worse outcomes [9]. Additionally, serum IL-6 is a strong indicator of the clinical severity of influenza virus-associated encephalopathy [10]. In an adult case series of ANE associated with COVID-19, increased serum and CSF IL-6 were found in some cases, especially in a case with RNA binding protein 2 (RANBP2) mutation, and seems to be associated with worse outcome [11]. RANBP2 is one of the main components of the nuclear pore complex and has numerous roles throughout the cell cycle [12,13]. Mutations in the RANBP2 gene are associated with ANE type1 (ANE1), where patients experience a sharp rise in cytokine production and hyperinflammation in response to viral infection, presumably by regulating distinct virus infection and innate immune response pathways [13]. A case-control association study with 31 ANE cases identified IL-10 polymorphism (rs1800781/rs1800782) as a genetic risk factor of ANE, which also supports that dysregulated immune response to viral infections plays a key role in the development of ANE [14].

The proposed mechanism of ANE shares a similar pathway with severe COVID-19 because a dysregulated immune response characterized by increased production of proinflammatory cytokines is considered to be a major driver. Negative CSF SARS-CoV-2 PCR and elevated serum and CSF IL-6 in the present case suggest that a dysregulated immune response contributed to the development of ANE and MOF. Although no evidence-based treatment has been established for ANE, immunomodulatory therapy, including corticosteroids, IVIG, and tocilizumab is empirically used to control cytokine production and signaling [15,16]. In addition, early TPE for ANE has recently been reported to remove circulating proinflammatory cytokines [17]. In a recent meta-analysis of case series, ANE treated with TPE had significantly better survival (100% vs. 45%, p=0.0001) [18]. In our case, the hemodynamics rapidly improved after TPE. Although a definitive causal relationship cannot be drawn from our single case experience, it might be possible that the removal of humoral mediators by TPE has contributed to the recovery of compromised circulation, as reported previously in multiple organ failure case series [19]. Reduction of the new production of cytokines by IVMP along with the removal of circulating humoral mediators by TPE is the theoretical background for this combination therapy. Our case was classified as high-risk (ANE-SS 6 points complicated with severe MOF) and likely to have moderate to severe sequelae or death (>90%) [13]. We presume that the combination therapy suppressed the cytokine production and signaling as indicated by decreased serum IL-6 after the treatment, which might have contributed to the complete neurological recovery. However, we highlight limitations to our study, including a single case report with no comparisons and incomplete measurements of serum and CSF proinflammatory cytokines as well as the invasive nature of TPE compared to other immunomodulatory therapies. The decision to introduce TPE should be carefully made case-by-case basis according to the facility’s proficiency.

Deterioration and recovery of EEG findings were delayed by several days following clinical multiple organ failure. This is consistent with a previous report of sepsis-associated encephalopathy [20], suggesting a complex interaction between systemic imbalance in homeostasis and the electrophysiology of the central nervous system.

## Conclusions

We reported a severe case of ANE with MOF following COVID-19 treatment with early immunomodulatory therapy and TPE with complete neurological recovery. Although further studies are needed, our case suggests that early immunomodulatory therapy and TPE may have contributed to the improvement in ANE and MOF.
